# Factors influencing treatment adherence in hypertension and HIV management in South Africa: A comparative literature review

**DOI:** 10.4102/safp.v64i1.5434

**Published:** 2022-06-29

**Authors:** Dimitra Enslin, Prabhakar Mallya

**Affiliations:** 1 Department of Health Sciences, Faculty of Life Sciences and Education, University of South Wales, Pontypridd, United Kingdom

**Keywords:** hypertension, adherence, compliance, HIV, counselling, health knowledge, patient education

## Abstract

**Background:**

Hypertension (HTN) is the most significant risk factor for cardiovascular disease (CVD) in South Africa (SA), with one in three people over the age of 25 suffering from HTN. Whilst human immunodeficiency virus and acquired immunodeficiency syndrome (HIV/AIDS) are the leading causes of death in South Africa, CVD is in the top 10 causes of death, demonstrating the importance of detecting and controlling blood pressure early on. This study aimed to review adherence factors to antihypertensive medication and antiretroviral therapy (ART) and evaluate the resulting factors influencing adherence discrepancies within the South African population.

**Methods:**

A comprehensive literature review was carried out. PubMed, ScienceDirect, Cochrane and Embase were searched for English publications between 2000 and 2021.

**Results:**

A total of 50 articles covering quantitative and qualitative studies were included. Many studies identified poor adherence levels to antihypertensive treatment, reaching a substandard adherence rate of 41.9%, whilst most studies on the HIV-positive population reported good levels of adherence, with adherence rates of more than 90%. Being of the male gender, advanced age, low socioeconomic status and a low level of education were associated with unsatisfactory adherence rates in both groups. Within the HIV group, more participants had better knowledge concerning the extent of their disease and its required treatments.

**Conclusion:**

The results present substandard adherence levels to antihypertensives compared with antiretroviral (ARV) adherence, despite the influence of more non-adherence factors in the HIV group. The authors recommend better adherence counselling for patients with HTN during every clinic visit, regular healthcare worker training and the implementation of ART adherence programmes in patients with hypertension.

## Introduction

Hypertension (HTN) is a prevalent global disease with the number of patients afflicted by it steadily increasing over the years. Since 1980, the number of individuals suffering from HTN has more than doubled, and it is estimated that by 2025 the burden of HTN may be present in more than 1.56 billion people worldwide.^1^ In South Africa, HTN has become the most significant risk factor for cardiovascular disease (CVD), with CVD remaining steadfast in the top eight causes of death.^2^ The prevalence of HTN in South Africa (SA) varies widely according to different studies, but it has been estimated that 46% of women and 44% of men suffer from HTN.^3^ Hypertension is the most significant risk factor for CVD, and the treatment of CVD places a significant burden on the limited resources of the South African health system. Thus, highlighting the importance of focusing on the prevention of CVD through proper medication adherence cannot be understated. By targeting prevention, the mortality and morbidity rates in SA should be improved, and it is through patient counselling that such changes can be brought to fruition.

One of the main issues regarding controlling blood pressure (BP) is patient medication adherence, with antihypertensive adherence at least 50% 1 year after treatment initiation.^4,5,6^ Adherence is defined by the World Health Organization (WHO) as ‘the extent to which a person’s behaviour – taking medication, following a diet, and executing lifestyle changes – corresponds with agreed recommendations from a healthcare provider’.^7^ Worldwide, chronic medication adherence is an issue, with many patients struggling to follow prescribed regimens because of the stress of side effects, poor understanding of the disease, substance abuse, socioeconomic status and polypharmacy, amongst many others.^7,8,9^

Adherence counselling has been an important topic in South Africa since the start of the global epidemic of human immunodeficiency virus and acquired immunodeficiency syndrome (HIV/AIDS), as SA has the highest number of people living with HIV (PLWH) globally, with an estimated 7.9 million people infected with HIV.^10^ To combat this staggering epidemic, SA launched an extensive nationwide antiretroviral therapy (ART) initiation campaign for anyone testing positive for HIV, focusing heavily on medication adherence.^3^ This extensive ART roll out increases the high levels of chronic disease within the South African population because PLWH live longer with the disease under better control; thus they are more at risk of developing other comorbidities.^3^ This in turn amplifies inadequate patient medication adherence. Furthermore, the public healthcare system is heavily overburdened and understaffed, resulting in overworked healthcare workers (HCWs) spending minimal time counselling and educating patients.

As a result of the high numbers of PLWH on ART, many studies have been undertaken to address medication adherence in this population group. A cohort study by Moosa et al.^11^ assessed the long-term adherence of ART by evaluating the ART counselling programmes and found that optimal long-term adherence was successful if these programmes were followed. There has been a greater emphasis on adherence and patient counselling in this population group than in any other in SA. It stands to reason that a similar focus on HTN education and counselling should improve adherence in patients. Therefore, the aim of this study was to review and compare literature on factors that influence adherence to antihypertensive and ART medications in South Africa. These findings should highlight how well patient counselling can affect adherence to medication and aid in alleviating some stigma towards HIV by emphasising adherence in HTN and showing the population that the diseases are treated with equal significance amongst HCWs. South Africa is unique because it faces a rise in chronic comorbidities whilst still battling a high HIV burden. If South Africa can improve adherence to antihypertensives despite the ongoing adherence issues with ART, then other countries who do not have such a high burden of HIV should be even more successful in improving adherence amongst people with HTN.

## Methodology

### Search strategy

A computerised search of PubMed, ScienceDirect, Cochrane and Embase was conducted to identify studies focusing on adherence to antihypertensives or ART in the South African population. The following search keywords and medical subject headings (MeSHs) were used: ‘adherence, non-adherence, compliance’ AND ‘hypertension, high blood pressure’ and ‘South Africa’. After that, a similar search was conducted using ‘adherence, non-adherence, compliance’ and ‘antiretrovirals, ARVs’ and ‘South Africa’. In addition, a manual search was conducted through the official online websites of various organisations and institutions and Google Scholar. These included the WHO, United Nations Programme on HIV and AIDS (UNAIDS), the South African National Department of Health (NDoH), the National Institute for Health and Care Excellence (NICE), the International AIDS Society (IAS) and the South African Hypertension Society. Once relevant articles were found, the abstracts were reviewed and full texts were retrieved. After that, citations from the various reference lists were examined and snowballing was implemented to find further articles on the topic.

### Eligibility criteria

Once the literature was found, the inclusion criteria tabulated in Table 1 were applied to narrow the search. The eligibility criteria are as follows: articles had to be in English, from peer-reviewed journals, and should focus on the South African adult population. Articles also had to focus on HIV and HTN only.

**TABLE 1:** Inclusion criteria.

Criteria	Definition
Time frame	2000–2021
Study setting	South Africa
Study design	RCT, cross-sectional, cohort, qualitative studies
Language	English
Article type	Full text available
Study population	Anyone above 18 years on either ART or antihypertensives only

ART, antiretroviral therapy; RCT, randomised ontrolled trial.

The selected articles could focus on the adherence issues with the relevant medication, patients’ understanding of their disease or the measures taken to improve adherence. Any article focusing on other countries, other comorbidities or written in languages other than English were excluded. If the article could not be found in the complete text, it was also discarded. Furthermore, guidelines and reports used had to have original data and specify the methods used to report their findings.

### Data collection and extraction

**FIGURE 1:**
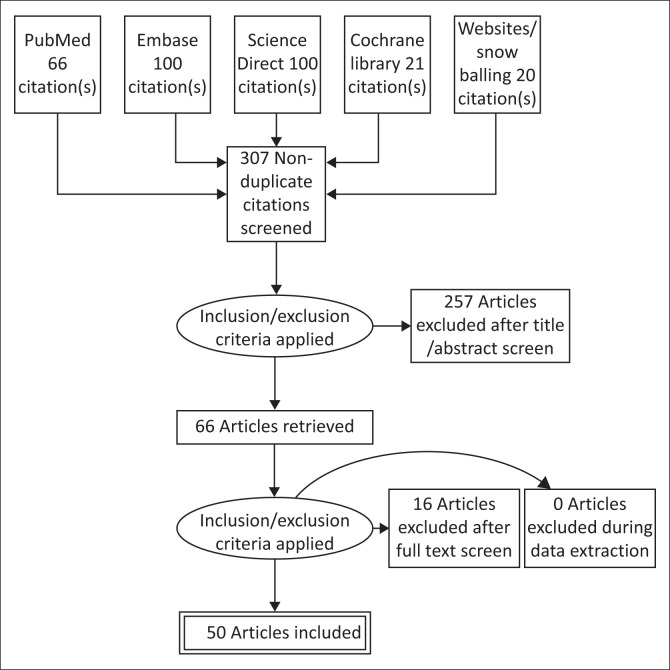
PRISMA flow chart of results.

All literature identified by the implemented search methods were tracked by listing the year, author and title in a word document to ensure no duplicates were used and keep track of all information gathered. After that each article was scrutinised by first screening the title, then reading the abstract and applying the inclusion and exclusion criteria. Then the full text was evaluated and highlighted if it should form part of the review. The Preferred Reporting Items for Systematic Reviews and Meta-Analyses (PRISMA) statement flow diagram in Figure 1 is used to demonstrate the study selection process. Study descriptions and summaries of main findings were compiled using Microsoft Word.

## Results

In each of the data bases searched, the first 100 titles were screened, using the time frame 2000 to 2021 as a filter. A total of 307 articles were identified, of which 257 were excluded after screening their full texts and applying the exclusion criteria. This yielded 50 records that fulfilled the criteria, resulting in 10 articles from PubMed, 17 from Embase, two from Science Direct and one from the Cochrane Library. An additional 20 articles were identified through websites and snowballing of references. Articles were excluded if the detailed studies were not conducted solely in SA, focused on other comorbidities or conducted on non-adult populations, and two articles were excluded as the full text was not available. This left a total of 50 articles for evaluation, with 20 articles focusing solely on HTN and adherence in SA and 30 articles focusing on HIV and adherence. A total of four studies were randomised controlled trials, 19 were cross-sectional, 20 were descriptive qualitative studies and seven were observational cohort studies. Looking at the study characteristics, 43 articles used their own data, whilst the rest analysed known data sets and one national guideline. With regard to gender, only three articles had a male majority population, whilst 53% had a more than 70% female majority study population. A total of 62% of articles had a young study population under the age of 50 years. Most of the study populations described an unemployment rate of > 50%, with most participants only having primary school education. Furthermore, 67% of studies focused on an urban population and only one study included private health sector patients.

Adherence levels were evaluated differently across studies. Apart from the RCT, most studies used semi-structured questionnaires and interviews to collect data and assess adherence. Only nine articles used validated tools to collect their data, with most researchers creating their own tools to gather adherence information. Five studies used viral load as an adherence marker, three studies used pill count and seven studies used both viral load and pill count with a semi-structured questionnaire to gather adherence information.

The factors influencing medication adherence were categorised into the following themes: level of adherence, health knowledge, education, finance, age and gender, stigma and social support, medication side effects and dosing, HCWs’ interactions with patients and counselling and current adherence to programmes.

**Figure 1** depicts this screening process in a PRISMA flow chart.

### Level of adherence

The identified studies assessed adherence in both the hypertensive and HIV-infected populations. Only Mafutha et al.^12^ found good adherence to antihypertensives amongst people with HTN, as 81% of participants adhered to their medication but did not comply with medical appointments or reduce their sodium intake. In contrast, most identified studies focusing on adherence to antihypertensives found adherence to medication to be inadequate.^13,14,15,16,17^ A cross-sectional study conducted in Sedibeng involving 251 participants showed that 16.7% of participants had controlled BP levels at the initial visit, which rose to 31.5% at their most recent visit.^14^ These findings demonstrated that participants who missed one, two or three doses per week had increased odds of uncontrolled HTN (OR: 3.57, 95% CI: 1.20–10.57; *p* = 0.02).^14^ Other studies proved that less than half of their study populations had their HTN under control.^15,18,19^ Adebolu et al.^13^ found that 41.9% of their study population adhered to their antihypertensives, whilst Rampamba et al.^20^ had an adherence rate of 54.6% amongst their study population.

In contrast, various studies on PLWH reported good levels of adherence to ART, with more than 90% adherence shown in their study populations.^11,21,22,23,24,25,26^ With regard to poor adherence, Van Dyk aimed to assess adherence issues after the national antiretroviral (ARV) rollout in SA in 2004 and found that only 40% of participants reached an optimum adherence level of above 90%.^27^ Another study found that only 56% of PLWH were on ART.^28^ Rabkin et al. highlighted the contrast that was found in this literature search with regard to adherence between the two disease groups by assessing relative compliance of individuals with both HIV and CVD. They assessed the CVD risk factors amongst adults attending an HIV clinic and demonstrated that whilst participants were adherent to their ART, most were not adherent to their antihypertensives.^17^

### Factors associated with poor adherence

#### Health knowledge and education

During this literature search, the authors found patients’ lack of knowledge regarding the extent of their disease and its management negatively affected adherence rates.^15,29^ Rampamba et al.^30^ used a pharmacist-led intervention to educate and counsel participants in the intervention group on HTN. Overall, they found that 90% of their participants did not have adequate knowledge about HTN management. The only statistically significant change post study intervention was the improvement in the understanding of what a normal BP is. This is echoed by Adebolu et al., who found that over 80% of their study population did not know what target BP they should be aiming for, yet they conceded that their results were not statistically significant (*p* = 0.585) when they cross-tabulated BP control versus knowledge of BP targets, suggesting that health knowledge is not a significant adherence factor or may be influenced by many other factors.^13^ Olowe et al. also found no association between the level of HTN knowledge and HTN control.^19^ Only Jongen et al. deemed their study population to have adequate or satisfactory knowledge of HTN.^31^

In the study by Rampamba et al., one of the significant associations found between participant characteristics and antihypertensive adherence was education level, as the proportion of adherent participants was much higher in the educated group (*p* = 0.036).^20^ Peltzer et al. found that 71% of their study participants, the majority of whom came from urban backgrounds, had education levels lower than secondary school learning, indicating that a low level of education is an issue across SA.^32^ However, these same authors found no link between education levels and HTN awareness and control, as did Thomas et al.^33^

The studies focusing on PLWH were divided. Many studies found that a low level of education and poor health knowledge negatively affected adherence to ARVs.^23,34,35^ Peltzer et al.^36^ found that higher levels of education were linked to a better knowledge of HIV, resulting in higher rates of HIV testing, which substantiated their theory that education levels could be viewed as an adherence factor. In contrast, both studies undertaken by Barry et al.^21^ and Dewing et al. found that poor educational levels and limited health knowledge did not primarily affect adherence, as those with a low level of education missed fewer clinic appointments and showed good adherence to ARVs (OR: 0.3, 95% CI: 0.1–1.1; *p* = 0.07), likely because the majority were unemployed and thus did not need to worry about taking off work to attend clinics.^37^ Furthermore, they agreed that although their participants’ adherence-related health information was sparse, it was not significantly associated with poor adherence, as factors such as finance influenced adherence much more.

Regarding health knowledge in PLWH, Terblanche et al.^38^ determined that most of their study participants knew very little about HIV, with a further 39% of participants noting that they had never received proper health education on their ART or HIV. Van Dyk highlighted worse adherence in participants who scored lower in ARV knowledge testing (*p* ≤ 0.0001).^27^ Again, one factor that arose in this study is that those participants who scored lower in the knowledge test also noticed that they did not always receive health education on their ARVs. The Southern African HIV Clinicians Society’s guidelines for ART in adults in 2020 also reported that a common reason for poor adherence in patients was HCWs not educating patients about the benefits of ARVs and not clearly explaining how medication should be taken.^39^ In contrast, several studies highlighted that most PLWH had a good knowledge of their disease and ARVs, which positively affected adherence.^26,40,41^ Nachega et al. also found that those within the nonadherent group were more aware of the detriments of missing an ARV dose (OR: 0.27, 95% CI: 0.08–0.91; *p* < 0.05) compared with those in the adherent group, which corroborates the theory that good knowledge does not necessarily result in good adherence.^41^

#### Finance

Financial insecurity is a common problem amongst most of the population in SA; thus, it is unsurprising that the results show finances as one of the major contributing factors to poor adherence in both the hypertensive and HIV-positive population.^12,21,23,25,31,34,37,40,42,43,44,45,46,47^ Three studies named cost of transport, cost of a healthy diet and cost of a workday lost as deterrents for treatment adherence in patients with HTN.^12,31,42^ Lack of food amongst some PLWH also increased the likelihood of treatment side effects and therefore undermined adherence, as patients on ARVs observed that they often did not have money for food and therefore could not take their treatment because of bad side effects when taken on an empty stomach (*p* = 0.003).^27,45^

#### Age and gender

Most studies found age and gender to influence adherence amongst both populations.^11,13,14,15,25,33,36,46,47^ Dewing et al. found gender to be significantly associated with nonadherence to ARVs (OR: 2.13, 95% CI: 1.37 to 3.30; *p* = 0.001), showing that males were 2.13 more likely to be non-adherent.^37^ With regard to age, the studies mentioned here found that the elderly tended to have worse rates of adherence, suggesting that forgetfulness or patient polypharmacy because of multiple comorbidities could be two influencing factors (OR: 0.27, 95% CI: 0.07–0.97; *p* = 0.045).^15^ Surprisingly, whilst Thomas et al.^33^ viewed gender as an adherence factor, they found no link between age and their study population’s adherence rate. Whilst Barry et al. noticed that participants above 50 did miss more clinic visits (OR: 2.02, 95% CI: 1.06–3.88; *p* = 0.03), they also found that this had no negative impact on patients’ adherence to ARVs and drug refills.^21^ Furthermore, Rampamba et al. (age: *p* = 0.422, gender: *p* = 0.574),^18^ Dalal et al.^48^ and Masilela et al. (*p* > 0.05)^18^ found no statistically significant association between gender or age and adherence to medication or control of BP.

#### Stigma and social support

Stigma was not addressed as an adherence factor amongst people with HTN, and only Dennison et al. mentioned the positive influence of having social support on antihypertensive medication adherence.^29^ With regard to the HIV-positive population, some participants described a good support structure through family and friends, which helped them come to terms with their disease and its treatment.^32,40,44,47^ In their study, Miller et al.^40^ mentioned that stigma did not influence ARV adherence, whilst Dahab et al.^34^ emphasised that good social support and belief in ARVs positively influenced adherence, whilst fear of HIV-related stigma negatively contributed to ARV adherence. Both Kheswa^45^ and Van Dyk^27^ listed stigma as a significant hindrance to ARV adherence.

#### Medication side effects and dosing schedules

Side effects of antihypertensives combined with dosing requirements had a negative influence on adherence in people with HTN. Adebolou et al. found a statistically significant association (*p* = 0.004) between side effects and BP control yet found there was no statistically significant association between taking multiple tablets a day and adherence (*p* = 0.527).^13^ Masilela et al. also found no significant changes in adherence to any dosing requirements.^18^ On the other hand, Duncan et al. noticed that adherence was affected when more than one antihypertensive was used (OR: 0.61, 95% CI: 0.39–0.96; *p* = 0.032).^15^

Conversely, in PLWH, far more studies listed side effects and dosing as a deterrent to good adherence.^22,25,27,34,43,47,48^ In Van Dyk’s study, participants with higher dosing schedules (*p* ≤ 0.0001) and higher pill burdens (*p* = 0.045) – defined as the number of tablets taken daily – showed lower levels of adherence to ARVs.^27^ Orrell et al. found three times daily dosing to negatively influence adherence to ARVs (relative risk [RR]: 2.0, 95% CI: 1.11–3.84).^25^ In contrast, Moosa et al. reported that despite a higher pill burden and dosing regimen, their participants still maintained reasonable adherence rates to their ART.^11^ Regarding side effects, only one participant in Miller et al.’s study^40^ mentioned side effects to be the reason for her discontinuation of her ARVs, whilst most participants in another study had no concerns about side effects from their ART.^41^

#### Healthcare workers’ patient interactions and counselling

None of the studies on HTN reported good HCW interactions with patients and counselling. Therefore, their influence cannot be accurately assessed. However, many noticed poor interactions and counselling to have negatively affected the adherence of their participants and emphasising ‘healthcare worker attitude’^31^ as a challenge to good adherence for many participants.^13,15,29,42^

Some studies focusing on the HIV-positive populations found HCW interactions to have a positive impact on ART adherence.^27^ The study by Moosa et al. showed successful treatment outcomes in 93.9% of participants, which in part was attributed to extensive pre-ART education and counselling sessions, continuous support during treatment and post-ART support programmes.^11^ In another study, 83.5% of participants indicated that their knowledge surrounding HIV and ART came from the HCW at their clinic, known as the ‘HIV sister’, suggesting a favourable relationship between patient and HCW.^38^ De Jager et al. reported improved adherence because of good patient counselling from HCWs (OR: 2.08, 95% CI: 1.24–3.5; *p* < 0.01).^22^

In addition, multiple studies on PLWH found poor HCW interaction and counselling to influence adherence to ART negatively.^38,45,46^ Both Penn et al.^47^ and Azia et al.^43^ listed communication and relationship between HCW and participant as a critical factor in adherence rates and disease management, with participants dissatisfied with services rendered often presenting with poorer adherence rates.

### Current adherence and management programmes

Many programmes aimed at improving adherence to both ART and antihypertensives have been implemented in South Africa with varying degrees of success. The following points show that a much bigger emphasis is placed on various adherence programmes for PLWH than for people with HTN.

#### Door-to-door visits

Using HCWs to go door-to-door within the community has proven to help monitor hypertensive patients and improve adherence, by reminding people to take their medication and using the visit for further adherence counselling.^49^ Such visits have also been recommended by Jongen et al. to raise awareness of HTN and ensure home measuring and adherence counselling.^31^ This comes after Doherty et al. had proven how effective door-to-door counselling and testing had been in the HIV-positive community (prevalence ratio [PR]: 1.54, 95% CI: 1.32–1.81).^50^

#### Mobile phones

Another programme found in both populations uses text messages or mobile phones to improve adherence by conveying reminders to take medication and visit the clinic regularly.^51,52,53^ After 1 year, Bobrow et al. showed an average decrease of systolic BP by –2.2 mmHg (95% CI: −4.4 to −0.04; *p* = 0.046) because of better medicine collection and adherence reminders.^51^ Furthermore, Leon et al. reported that one-third of their participants had improved attitudes towards HTN and showed improved adherence rates to antihypertensives following the text message reminders.^52^ Overall, they found this type of programme to be beneficial and acceptable to the community.

Madhavani et al. ran a similar study to determine whether mobile phones could be used as a reminder tool in PLWH.^53^ They found that 48.8% used their phone as a medication reminder device but only 10.5% of participants used their mobile phone as an appointment reminder. Their results suggest that a single prompt during each scheduled medication dose may improve adherence. However, they did identify that women over the age of 35 years (*p* = 0.012) with low levels of education (*p* = 0.011) and a low socio-economic status (*p* = 0.014) were not using mobile phones, indicating that using this as a reminder tool might not be possible across all populations. The reasons for this vary between inability afford a mobile phone or data, not willing to use one and not understanding how to use one.

#### Educational interventions

Magadza et al.^54^ investigated the effect of an educational intervention on HTN knowledge and adherence made up of presentations, monthly meetings and information leaflets. This led to increased awareness of HTN (*p* < 0.0001) and decreased concerns regarding medication side effects (*p* < 0.01). There was also an overall increase in adherence after the educational sessions, although it was not statistically significant enough (*p* > 0.05).

The use of visual aids to improve adherence has also been studied, mainly in the HIV-positive population. Wong et al. developed an educational video to improve medication-taking and found a statistically significant increase in participant knowledge (*p* = 0.021).^55^ Jones et al. studied how the use of active visualisation can affect adherence to ART by measuring viral loads before and after the educational intervention and reported better viral loads (*p* = 0.06) and follow-ups (*p* = 0.028) in the intervention group, showing that even brief visual interventions can improve adherence.^56^ They support the use of visualisation as an alternative for educating patients, although it was noticed that the use of viral loads may have been a limitation because resistance tests were not carried out; thus, it could not be concluded that differences in viral loads were exclusively because of improved adherence. Using an illustrated information leaflet on ARV side effects, Browne et al. studied what impact such illustrations could have on ARV knowledge.^57^ Side effect knowledge increased significantly in the intervention group (*p* < 0.0001), and participants found the leaflet clear and informative.^57^ The authors found that this is a simple yet efficient way to improve knowledge, even in patients with low levels of education, and saves time in busy, understaffed clinics. By teaching participants about side effects, their adherence should improve as they know what to expect.

#### Adherence clubs

The formation of adherence clubs (ACs) for PLWH is another programme aiming to improve adherence.^22,28,37^ Participants attending AC were more satisfied, having a shorter waiting time than those attending clinics (*p* < 0.001)^22^ and showed improved adherence because of improved satisfaction (OR: 2.08, 95% CI: 1.24–3.5; *p* < 0.01).^22^ Whilst challenges such as access to resources, finances and having a good relationship with clinics are issues for an AC, they are still seen as beneficial as they lead to stronger communities, patient self-empowerment and better peer support whilst having the added benefit of generating greater awareness of HIV and decreasing the stigma surrounding it.^28^ No ACs have been implemented or researched in people with HTN.

#### Fast-track treatment initiation counselling

Fast-track treatment initiation counselling (FTIC) with ART is a significant programme currently being implemented in SA. Comparing FTIC to standard care showed a 6% increase in ART initiation associated with FTIC (risk differences [RD]: 6.3%, 95% CI: −0.6% to 13.3%) but no reductions or improvements in viral load suppression and long-term retention.^58^ Wilkinson et al.^59^ supported the use of FTIC, as it helps in counselling patients from an early stage and emphasises the importance of good adherence. In their study, only 3.6% of participants were lost from care after adapting ART initiation and counselling, and they achieved significantly higher rates of viral load suppression, partly because of the educational and motivational interviewing received earlier than usual. Furthermore, Wilkinson et al. reported rapid initiation and counselling to be a feasible solution in overcoming adherence factors and strengthening treatment support.^59^ Fast-track treatment initiation counselling and AC are both recommended in the adherence guidelines by the NDoH.^60^

## Discussion

It is well known that adherence to antihypertensives is a significant problem worldwide, yet very few studies have focused on the South African population and how it manages adherence. The authors reviewed and compared studies that investigated factors influencing adherence to antihypertensive and ARV treatments in SA. The burden of both diseases is exceptionally high in SA, with the South African Demographic and Health Survey (SADHS) reporting a HTN prevalence of 46.0% in females and 44.0% in males above 18 years of age, with over 80% of hypertensive persons having uncontrolled BPs.^61^ The South African prevalence is higher than the overall global prevalence of HTN at 31.1%.^62^ Contrastingly, the SADHS reported the prevalence of HIV in 21.2.% of all South Africans between 15 and 49 years and in 13.0% of people over 50 years, with 62.0% of all PLWH in South Africa on treatment and 54.0% virally suppressed.^63^ Whilst both diseases are not well controlled, the figures show that HTN is more prevalent and has a much higher number of poorly controlled persons.

Whilst many studies have been undertaken to evaluate common adherence factors such as finance, side effects, dosing frequencies, gender and age, there are limited studies in SA focusing on patient counselling and educating them on their disease, especially in people with HTN. This review found various modifiable factors contributing to non-adherence in the hypertensive and HIV-positive populations, including health knowledge,^15,27,28,30,38^ level of education,^20,23,34,35,36^ finance,^12,21,23,25,31,34,37,40,43,44,45,46,47^ stigma,^27,34,45^ dosing frequencies^15,22,27,34,43,48^ and HCWs’ interactions with patients.^13,14,15,28,31,38,39,42,43,46,47,64^ Both population groups are affected equally by age, gender, finance, dosing frequency and level of education. The differences are that PLWH must deal with a higher burden of medication side effects and stigma within their community, whilst hypertensive persons suffer from worse HCW counselling, poor health knowledge and a higher pill burden.^15,22,25,27,34,43,47,48^

Lack of disease knowledge was prevalent in both populations but more so in the HTN group and thus contributed to worsening adherence.^13,15,29,30^ Overall, PLWH did show better knowledge about their disease when compared with people with HTN, underlining part of why PLWH seem to have better adherence rates.^26,40,41^ This is in keeping with results from other studies in various countries that showed that poor understanding of the severity of HTN was associated with nonadherence.^4,65^ People living with HIV have better health knowledge because of better, more frequent counselling, which leads to higher adherence rates, underscoring the importance of adequate health education amongst patients with chronic diseases. Health knowledge is one factor amongst people with HTN that can be modified by HCWs and needs to form an integral part of every clinic visit. A Turkish RCT by Hacihasanoğlu et al. evaluated the effect of patient education on adherence and BP control and saw a successful reduction in BP and better adherence to their interventions, which consisted of nurses giving six monthly education sessions, education during clinic visits and two home visits.^66^ This is a practical education model that HCWs in SA could implement to promote patient education. Another feasible concept that may improve HTN awareness and control in SA is moving health education away from medical settings to other vital settings in the community such as barbershops, churches and grocery stores.^67,68,69^ Multiple overseas studies have shown improved awareness, health behaviour and better BP control through such measures.^67,68,69^

Furthermore, side effect profiles of antihypertensives are less pronounced compared with ART.^13,22,34,43,48,70^ Whilst this may positively affect antihypertensive medication adherence, one must remember that the low side effect profile and silent nature of the disease may contribute to poor adherence, as often minimal change can be seen with good adherence.^15,20^ Whilst ARV side effects are far more common and play a major role in poor adherence, the advancement in the development of new drugs has slowly decreased their side effect profiles and increased their tolerability and acceptance.^70^ Being compliant with ARVs leads to a longer life expectancy, making their effects more obvious compared with antihypertensives.^71,72^ Despite the ARV side effects, adherence rates remain higher in PLWH compared with people with HTN (changed from the hypertensive population), indicating that other factors may play more influential roles in improving adherence.

One such adherence factor is how HCWs interacted with the different population groups. Most of the studies reported good HCW interactions with PLWH, stating that they were often given enough information during clinic visits.^11,22,27,38,46^ Unfortunately, the authors did not find similar results for the people with HTN, as none of the hypertensive studies reported good HCW interactions and counselling with their study participants.^13,15,29,31,42^ The lack of emphasis on patient–HCW interaction in the HTN group is in stark contrast to the HIV-positive group, highlighting that the training of HCWs is different depending on the disease and that many people do not realise the necessity of adherence counselling in people with HTN.^11,22,23^ This is not helped by the fact that HTN is a symptomless disease, with many patients not treating it as a serious comorbidity and thus being less likely to tolerate high pill burdens when they cannot see the necessity for it. HCWs need to address such beliefs and find ways to adjust patients’ thoughts on HTN by being more involved in adherence counselling at every clinic visit.

In SA, there are far more measures taken to manage HIV than HTN.^28,37,55,56,57,58,59,60^ The authors only found three management programmes for the HTN population, on top of the standard doctor’s visit, and those programmes are also used in PLWH: HCWs going door-to-door, using text messages to promote adherence and pharmacist-led counselling, of which the first two had a statistically significant impact on medication adherence.^30,31,49,50,51,52,53^ There are more programmes for PLWH, including ACs, using visual aids and educational material and FTIC.^28,37,55,56,57,58,59,60^ All these programmes are aimed at improving adherence and initiating as many positive patients as possible on ART. Such programmes are not utilised in South Africans with HTN, who could benefit significantly from such measures; one reason could be that HTN is considered a silent disease, making it challenging to convince patients of its severity.

This narrative review identified some concerns with regard to the studies involved. Firstly, in most studies, the population used was not representative of the general population as they were often a small sample size, skewed towards female majority, younger people under the age of 50 years and unemployed and uneducated people. Whilst this does reflect the socio-economic issue SA faces, it makes it harder to apply the results to all South Africans. As most participants were recruited from local clinics, it is unsurprising that females are the majority, as they have better health seeking behaviours than men and tend to be unemployed, as men are most commonly the breadwinners and, thus not always able to attend clinics. Secondly, different tools were used to assess adherence in the various studies, making it difficult to rely on the data and showing uniformity. Adherence is notoriously difficult to measure as it is influenced so much by a person. Attempts have been made to measure it directly through pill counting, viral load changes and clinic appointments visited in eight studies, thereby trying to lessen the influence of bias that comes with self-reporting on adherence.^11,21,23,24,25,34,51,54^ However, the issue with using viral load is that one cannot exclude treatment failure and resistance to medication rather than poor adherence as the reason for high viral loads. Furthermore, pill count does not consider lost pills and can be easily manipulated by the patient. Most of the studies used relied solely on self-reported adherence through questionnaires and interviews, which may be subject to self-presentation and recall bias, often overestimating the effects of the intervention being tested.^65^ However, such self-reflection can give good insight into adherence factors and interventions that may have not been included previously. Using interviews may also make it difficult not to influence the participant’s answers and probe in the direction of the study goals, but again allows for more insight into an individual’s thought process regarding adherence. Lastly, it is concerning that only nine studies used validated questionnaires and tools to gather their data.^12,15,16,18,19,22,26,29,41^ Using such tools makes it generalisable not only to SA but to the global population as such tools have been tested in different populations.

Despite PLWH having their unique non-adherence factors, they still have better adherence rates compared with people with HTN.^11,13,20,21,22,24,26,61^ This highlights the significant influence of good patient counselling and disease education on adherence and showcases the gap in managing HTN in SA. Good HCW interactions and counselling of patients must become the cornerstone of managing hypertensive patients. This can be carried out by implementing adherence counselling in popular community settings and adapting some HIV programmes for people with HTN to see if they can improve adherence.

### Study limitations

One limitation of this review is that the various studies used different methodological qualities, which may have had an impact on the described results and biased the findings and interpretations. Furthermore, only peer-reviewed studies in English were included, which may have restricted the results.

## Conclusion

In conclusion, compared with HTN, there is a larger pool of studies on factors affecting treatment adherence in PLWH, signifying the need for more studies to be conducted on treatment adherence amongst patients with HTN. This also reflects that more care is being taken in managing PLWH and emphasising adherence to ART. This review highlights that counselling and patient education positively influenced treatment adherence in PLWH, despite the added obstacles this population group faces, which calls for better adaptation of these activities into HTN treatment programmes in SA. To this end, HCWs need to be empowered with skills to implement these adaptations. Future studies should assess the feasibility and effectiveness of implementing HIV adherence programmes in people with HTN by focusing on larger, more generalised sample populations, using validated questionnaires and using multiple, different techniques to collect data on adherence.
